# Using large language models to identify prediagnostic clinical features of ovarian cancer from healthcare records: a population-based case–control study

**DOI:** 10.3399/BJGP.2025.0366

**Published:** 2026-06-16

**Authors:** Garth Funston, Namu Park, Matthew Thompson, Meliha Yetisgen, Barbara A Goff, Larry Kessler, Fiona M Walter

**Affiliations:** 1 Centre for Cancer Screening, Prevention and Early Diagnosis, Wolfson Institute of Population Health, Barts and The London School of Medicine and Dentistry, Queen Mary University of London, London, UK; 2 Department of Biomedical Informatics and Medical Education, University of Washington, Seattle, WA, US; 3 Department of Family Medicine, University of Washington, Seattle, US; 4 Department of Obstetrics and Gynecology, University of Washington, Seattle, WA, US; 5 Department of Health Systems and Population Health, School of Public Health, University of Washington, Seattle, WA, US; 6 The Primary Care Unit, Department of Public Health and Primary Care, University of Cambridge, Cambridge, UK

**Keywords:** cancer, early diagnosis, large language models, ovarian, primary health care

## Abstract

**Background:**

Most women with ovarian cancer are diagnosed after developing symptoms. However, symptoms are often recorded as free text within electronic health records (EHRs), which is not readily accessible for research.

**Aim:**

To use EHRs to examine associations between coded and large language model (LLM)-extracted free-text clinical features with ovarian cancer diagnosis.

**Design and setting:**

Population-based case–control study using EHRs and cancer registry data from women attending primary care, outpatient, and emergency clinics associated with the University of Washington, US.

**Method:**

In total, 136 women with ovarian cancer cases (diagnosed 2012–2019) were matched (age, clinic type) to 1360 control participants. Twelve months of prediagnosis coded and free-text data were extracted from EHRs. LLMs were tested on annotated notes, before extracting information on 17 prespecified clinical features. Univariate conditional logistic regression analyses were used to identify clinical features associated with ovarian cancer.

**Results:**

There were 14 clinical features that were more commonly identified from free text using LLMs than from codes in both the case and control groups. There were 14 features that were significantly associated with ovarian cancer when using codes and LLM-extracted data, but only eight features were significant using codes alone. Using both coded and LLM-extracted data, 11 features had odds ratios >2. Thirteen features were significantly associated when restricting analysis to early-stage (I–II) diagnosis.

**Conclusion:**

There is an identifiable ovarian cancer symptom signature within EHRs, with LLM-based natural language processing approaches enabling extraction of key non-coded symptom information. LLMs could support EHR-based research, while LLM-based clinical decision support tools may improve identification of patients with symptoms of possible cancer.

## How this fits

Most women with ovarian cancer present with symptoms, but many symptoms are recorded only in free-text healthcare records and missed by studies and clinical decision support tools that rely on coded data. This study found that using large language models (LLMs) to extract symptoms from free-text records substantially increased symptom detection and strengthened associations with ovarian cancer. Incorporating LLM-extracted symptom information into research and clinical decision tools may support identification of women at higher risk of cancer and aid appropriate investigation.

## Introduction

Ovarian cancer is the most lethal form of gynaecological cancer. The 5-year survival rate is 48% in the US and even lower in many countries.^1,2^ Outcomes are highly dependent on stage at diagnosis, with 5-year relative survival rates of 92% for women with stage I, compared with 32% with stage IV ovarian cancer.^3^ Given the potential importance of early-stage diagnosis, large screening studies have been conducted but have not demonstrated a reduction in mortality.^4,5^ In the absence of screening programmes, most women are diagnosed after presenting with symptoms in primary care, gynaecology, or to emergency department settings.^6–8^ Research has found 80% of women report having symptoms 3–12 months before diagnosis and symptoms can occur in both early- and late-stage disease.^9^ However, symptoms are often non-specific, so clinicians may not suspect ovarian cancer and patients frequently present multiple times before the disease is suspected and a diagnosis made.^10^ There may be a window of opportunity for more timely detection and treatment initiation of ovarian cancer if symptoms are recognised and investigated earlier.

A review published in 2020 identified over 20 symptom-based tools that have been developed to help guide clinicians in assessing women for possible ovarian cancer.^11^ Studies have used a variety of approaches to identify symptoms and signs that may be associated with ovarian cancer, including patient surveys, hand reviews of medical notes, and use of clinical codes.^11^ Hand review of clinical notes is a ‘gold-standard’ method but is highly resource intensive, whereas relying on clinical codes is limited by the fact that around 80% of medical record information is recorded in unstructured (‘free text’) rather than coded format.^12^

Natural language processing (NLP) is a subfield of artificial intelligence that is increasingly being used to identify clinical features recorded within the unstructured sections of electronic health records (EHRs).^13^ This technique has the potential to significantly improve the quantity and granularity of symptom data that can be extracted from the EHR, particularly given the growing capabilities of large language models (LLMs). The aim of the study was to use LLMs to extract prediagnostic clinical features from free-text medical records and, along with coded data, compare their frequency among women with and without ovarian cancer presenting in the primary care, outpatient, or emergency medicine setting.

## Method

### Study design

A case–control design was adopted using EHR data from University of Washington Medicine (UWM) linked to the Seattle/Puget Sound Surveillance, Epidemiology, and End Results (SEER) Program. This registry is funded by the National Cancer Institute to ascertain all incident cases of cancer (excluding non-melanoma skin cancer) in the 13-county region around metropolitan Seattle, Washington, US.

### Setting

Case and control participants were identified from women who received ambulatory care at UWM, an academic health science centre serving western Washington state (US).

### Participants

Case participants were defined as women aged ≥18 years with afirst primary ovarian cancer diagnosis recorded in the Seattle/Puget Sound SEER registry between 1 January 2012 and 31 December 2019 (Supplementary Tables S1 and S2).

For inclusion in the analytic cohort, case participants were additionally required to have an established ambulatory care relationship within UWM in the 2 years before diagnosis and evidence of diagnostic evaluation for possible ovarian cancer documented in the EHR, defined as an imaging investigation (computed tomography or ultrasound) and or referral initiated from an ambulatory clinic encounter.

An ambulatory care relationship was defined as ≥1 encounter in one of four clinic categories:

- primary care, which includes family medicine, internal medicine, international medicine, and urgent care;
- women’s health, which includes obstetrics and gynaecology;
- emergency medicine; and
- other, which includes all other ambulatory clinic types.

Individuals in the case group required EHR evidence of an investigation and/or referral for possible ovarian cancer initiated at that clinic. Ovarian cancer diagnosis was verified through linkage to regional SEER.

Eligible control participants were randomly selected using a 10:1 matched sample. Women were eligible for control group selection if their date of birth was within 3 years of the case participant, they had ≥1 visit to the same clinic type (categorised above) as the case participant, had no history of ovarian, fallopian, or peritoneal cancer (based on the absence of International Classification of Diseases [ICD] codes in their EHR) (Supplementary Table S2), and no history of bilateral salpingo-oophorectomy. The date of visit to the same type of clinic as the case participant served as the index or ‘lookback’ date for data extraction. For control participants who had multiple eligible visits, the earliest visit within the 12-month observation window was selected as the index date.

### Data collection

The UWM enterprise-wide data warehouse was used to obtain EHR data. The warehouse provides a central repository that integrates EHR across the UWM healthcare system. EHR data were obtained for all encounters in the 12-month period before the diagnosis date (case group) and index date (control group). This corresponded to a total of 4564 clinical reports for the case group (mean 50.7 reports per case participant) and 41 384 reports for the control group (mean 33.8 reports per control participant). Data obtained from SEER for the case group included ovarian cancer histology and stage at diagnosis.

### Symptom extraction

The ICD-10 codes for 17 possible ovarian cancer-related symptoms, identified from the literature,^11,14–16^ were extracted from coded EHRs (Supplementary Table S2). To extract symptom information from free-text EHRs, this study used a publicly available LLM, Llama 3.1-8B (Meta) and a proprietary counterpart, GPT-4o (OpenAI). A subset of the dataset, consisting of 100 clinical reports from patients with ovarian cancer, was annotated by medical students for the presence or absence of the 17 symptoms.

LLM evaluation was conducted using two prompt designs. Prompt 1 included task instructions, a list of target symptoms, and the desired output format (Supplementary Information S1). Prompt 2 extended prompt 1 by adding a list of synonyms for each symptom to enhance the sensitivity of extraction (Supplementary Information S2). In both prompt designs, the model was instructed to extract only symptoms that were explicitly stated in the clinical text. Explicit mentions were defined as symptoms directly described in the report, regardless of word order or phrasing (for example, ‘nausea and vomiting’, ‘vomiting and nausea’, or ‘the patient reports nausea and later vomited’). In contrast, symptoms inferred from related but non-explicit descriptions, such as predicting nausea from phrases like ‘poor appetite’, were considered errors. This explicitness requirement was intended to reduce mispredictions arising from inferred reasoning rather than documented evidence. All experiments were conducted on a HIPAA-compliant server. LLM inference was performed with temperature set to 0 and top_k set to 1 to minimise stochasticity. Repeated spot checks showed negligible variation in extracted symptoms and no meaningful impact on downstream F1 scores.

Based on evaluation set performance, GPT-4o with prompt 2 achieved the highest overall results, reaching a micro-averaged F1 score—a metric that balances precision and recall across all classes—of 0.83 (Supplementary Table S3). Given its strong performance, GPT-4o was selected to process the entire dataset. For this full-scale application, a hybrid prompting strategy was employed by selecting the best-performing prompt for each symptom individually. Specifically, prompt 1 yielded higher F1 scores for symptoms such as pelvic mass, urinary frequency, fatigue, nausea, and weight loss, and was used to extract information on those symptoms. For all other symptoms, outputs generated using prompt 2 were selected. Although prompt 2 improved sensitivity for several symptoms, subsequent error analysis indicated that expanded synonym lists could introduce overinference for certain features. In particular, broader related terms occasionally increased false-positive symptom identification when symptoms were not explicitly documented.

For model evaluation, a subset of 100 clinical reports from patients with ovarian cancer was manually annotated for the presence or absence of the 17 target symptoms by two trained medical students, under the supervision of a clinician. These annotations served as the gold-standard labels against which LLM-generated outputs were compared to compute the quantitative performance metrics reported in Supplementary Table S3. In addition, a qualitative error analyses was conducted to examine discrepancies between LLM predictions and human annotations, including missed symptoms and false positives. Exhaustive manual validation was not feasible across the whole dataset. Instead, targeted sanity checks were performed on a randomly selected subset of extracted reports to confirm that symptom extraction patterns were consistent with those observed in the annotated evaluation set.

### Data analysis

The characteristics of the case and control groups including age, race/ethnicity, and number of consultations in the 12 months before diagnosis were summarised. The frequency of symptoms and signs obtained from coded data was compared with that extracted from free-text data overall in the case and control groups using descriptive statistics. The proportion of the case and control groups with each symptom/sign in the 12 months before diagnosis were compared using conditional logistic regression to calculate odds ratios (ORs) and 95% confidence intervals (95% CIs).

Statistical analyses were conducted using Python 3.9 with the packages SciPy (version 1.4.1) and Statsmodels (version 0.14.4).

## Results

### Participants

**Figure 1.**
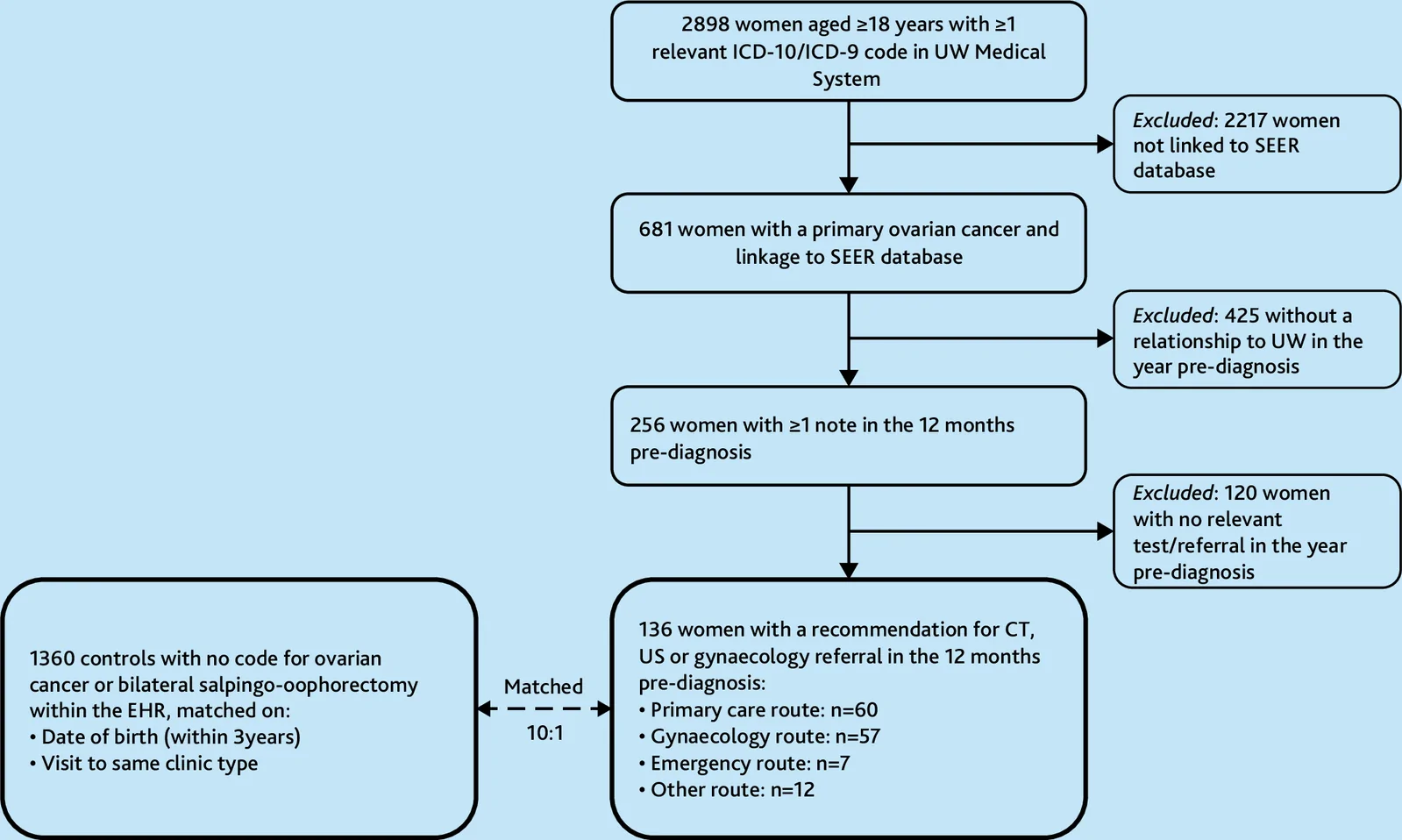
Case and control participant selection. Case participants were defined by confirmed ovarian cancer diagnosis in the SEER registry. Additional inclusion criteria, including evidence of diagnostic investigation or referral in ambulatory care records, were applied to ensure availability of relevant prediagnostic clinical information for analysis. CT = computed tomography. EHR = electronic health record. ICD = International Classification of Diseases. SEER = Surveillance, Epidemiology, and End Results. US = ultrasound. UW = University of Washington.

A total of 2898 women with possible ovarian cancer were identified from EHRs (Figure 1). After linkage with the cancer registry, 681 had evidence of a primary ovarian cancer, of whom 256 met criteria of an ambulatory care relationship with UWM in the 2 years before their date of diagnosis. A further 120 patients were excluded who lacked evidence of an imaging test or gynaecology referral recommendation at UWM during this period. The final sample included 136 case participants matched to 1360 control participants.

### Description of case and control groups

The mean age was 60.7 years (standard deviation [SD] 13.03) for case participants and 60.3 (SD 13.19) for control participants (Table 1). The most common ethnicity among the case and control groups was White (76.5% [104/136] and 68.2% [927/1360], respectively). Within the 12 months before diagnosis, the case group had a median 13.0 clinical health care visits, compared with 7.0 for the control group. The stage distribution of the 136 case participants was as follows: stage I: 30 (22.1%), stage II: 11 (8.1%), stage III: 70 (51.5%), stage IV: 16 (11.8%), and stage missing: 9 (6.6%).

**Table 1.** Characteristics of women with ovarian cancer (case group) and control group in ambulatory care

Characteristic	Case group(*N* = 136)	Control group(*N* = 1360)
**Age, years, mean (SD)**	60.7 (13.03)	60.3 (13.19)
**Race/ethnicity**		
White	104 (76.5)	927 (68.2)
Asian	13 (9.6)	108 (7.9)
African American	8 (5.9)	95 (7.0)
Other/unknown	7 (5.1)	188 (13.8)
White Hispanic	4 (2.9)	42 (3.1)
**Number of clinical visits before diagnosis/index date, median (IQR)**	13.0 (8.0–25.2)	7.0 (3.0–17.0)

Data are *n* (%) unless otherwise specified. IQR = interquartile range. SD = standard deviation.

### Frequencies of clinical features from coded and free-text data

Abdominal mass was the only clinical feature more frequently identified through ICD codes than free-text data among the case group, whereas pelvic mass and pelvic pain were more frequently captured through codes than free-text data in the control group (Table 2). Across all other features (14 features), extraction from free-text clinical notes yielded higher frequencies than ICD codes. Combining coded with LLM-extracted features resulted in higher overall frequencies across all features, indicating that each method contributed independently to clinical feature identification. For the case group, an additional 41.9% (57/136) of the case group were identified as having abdominal distension/bloating, 40.5% (55/136) as having fatigue, and 38.2% (52/136) as having abdominal pain when LLM-extracted symptoms were used in addition to coded symptoms.

**Table 2.** Frequency of coded and large language model-extracted clinical features in case and control groups

	Case group (*N* = 136)*n* (%)			Control group (*N* = 1360)n (%)		
**Clinical feature**	**Identified from coded data**	**Identified from free text**	**Identified from either coded or free text**	**Identified from coded data**	**Identified from free text**	**Identified from either coded or free text**
**Abdominal**						
Ascites	12 (8.8)	25 (18.4)	30 (22.1)	16 (1.2)	19 (1.4)	29 (2.1)
Abdominal distention/bloating	14 (10.3)	70 (51.5)	71 (52.2)	43 (3.2)	248 (18.2)	266 (19.6)
Abdominal mass	60 (44.1)	58 (42.6)	82 (60.3)	35 (2.6)	64 (4.7)	89 (6.5)
Abdominal pain	40 (29.4)	87 (64.0)	92 (67.6)	172 (12.6)	351 (25.8)	429 (31.5)
Change in bowel habits	26 (19.1)	73 (53.7)	78 (57.4)	213 (15.7)	415 (30.5)	497 (36.5)
Early satiety	3 (2.2)	33 (24.3)	34 (25.0)	7 (0.5)	53 (3.9)	58 (4.3)
Loss of appetite	1 (0.7)	49 (36.0)	49 (36.0)	17 (1.3)	196 (14.4)	200 (14.7)
**Pelvic**						
Pelvic mass	58 (42.6)	66 (48.5)	84 (61.8)	33 (2.4)	27 (2.0)	53 (3.9)
Pelvic pain	40 (29.4)	42 (30.9)	64 (47.1)	215 (15.8)	140 (10.3)	299 (22.0)
Vaginal bleeding	20 (14.7)	36 (26.5)	38 (27.9)	88 (6.5)	224 (16.5)	254 (18.7)
**Urinary**						
Dysuria	12 (8.8)	20 (14.7)	27 (19.9)	147 (10.8)	196 (14.4)	286 (21.0)
Urinary frequency	19 (14.0)	44 (32.4)	51 (37.5)	111 (8.2)	200 (14.7)	248 (18.2)
Urinary incontinence	7 (5.1)	24 (17.6)	29 (21.3)	94 (6.9)	201 (14.8)	242 (17.8)
Urinary urgency	3 (2.2)	25 (18.4)	25 (18.4)	45 (3.3)	155 (11.4)	177 (13.0)
**Non-specific**						
Fatigue	32 (23.5)	80 (58.8)	87 (64.0)	248 (18.2)	490 (36.0)	572 (42.1)
Nausea	21 (15.4)	63 (46.3)	68 (50.0)	131 (9.6)	425 (31.3)	469 (34.5)
Weight loss	5 (3.7)	47 (34.6)	47 (34.6)	53 (3.9)	283 (20.8)	303 (22.3)
**Summary**						
Any clinical feature (≥1 of 17 symptoms)	102 (75.0)	126 (92.6)	131 (96.3)	629 (46.3)	820 (60.3)	931 (68.5)

Data are *n* (%) unless otherwise specified.

Among the case group, the proportion of women with ≥1 documented clinical feature increased from 75.0% (102/136) using coded data alone to 92.6% (126/136) using free-text alone and 96.3% (131/136)when combining both sources (Table 2). In contrast, although symptom coverage also increased among the control group when free-text extraction was applied, overall proportions remained substantially lower than in the case group (46.3% [629/1360] with coded data alone, 60.3% [820/1360] with free-text alone, and 68.5% [931/1360] when both sources were used).

### Association of clinical features with ovarian cancer

**Figure 2.**
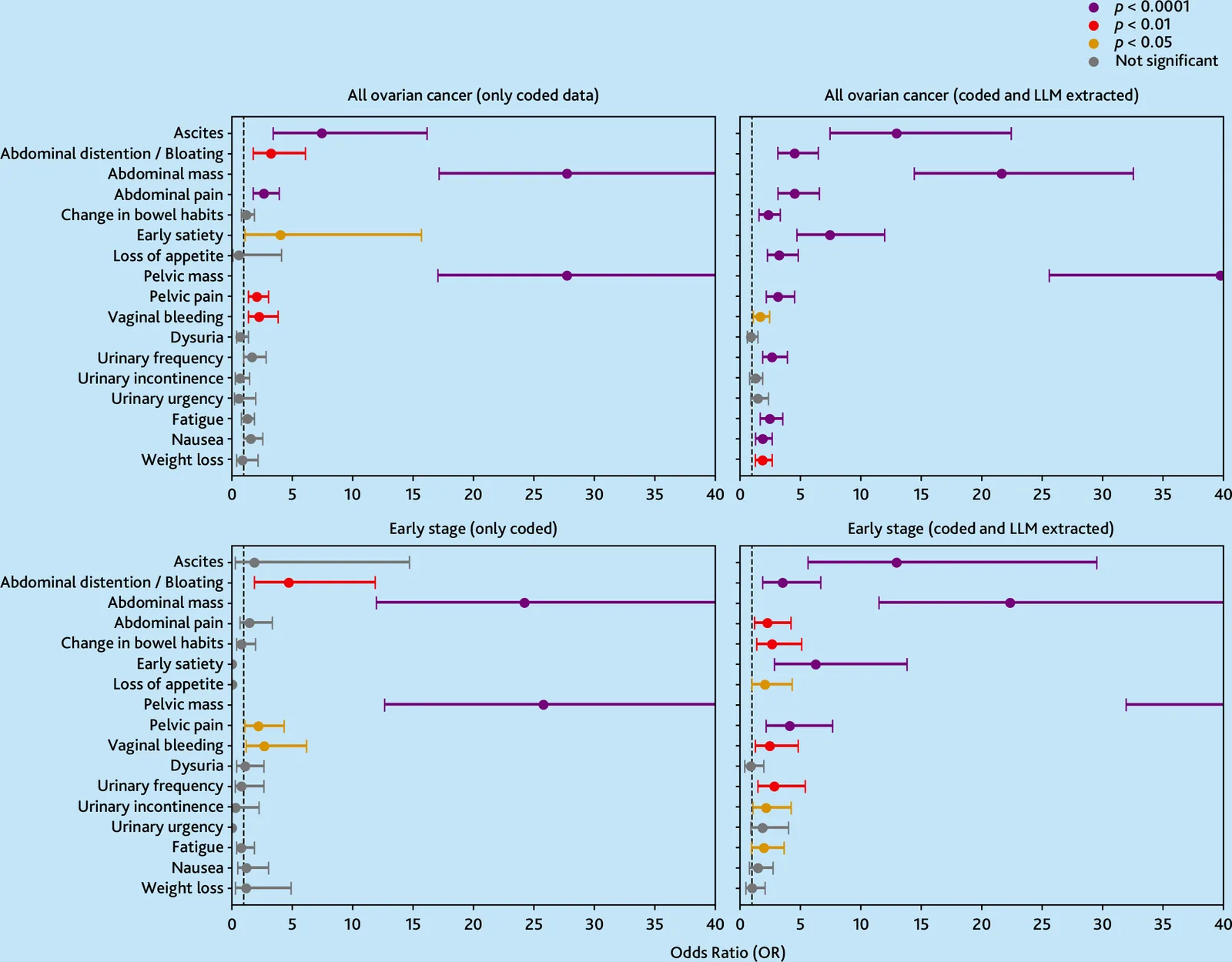
Associations between coded and large language model (LLM)-extracted clinical features with ovarian cancer and early-stage (I–II) ovarian cancer. Data are odds ratios (ORs) and 95% confidence intervals (CIs). Pelvic mass OR for early stage (coded and LLM extraction not shown OR = 67.3, 95% CI = 32.0 to 141.4, see Supplementary Table S4).

When using coded and LLM extraction, 14 out of 17 clinical features were significantly more common in the case than the control group (Figure 2). ORs were >2.0 for ascites, abdominal distension/bloating, abdominal mass, abdominal pain, change in bowel, early satiety, loss of appetite, pelvic mass, pelvic pain, urinary frequency and fatigue. Dysuria, urinary *incontinence* and urinary urgency were not significantly associated with ovarian cancer.

When the analysis was restricted to coded data, 8 of the 17 clinical features remained significantly associated with ovarian cancer (Figure 2). With the exception of abdominal mass and vaginal bleeding, ORs were smaller when using coded data alone. The six clinical features no longer associated with ovarian cancer when using coded data alone were change in bowel habits, loss of appetite, urinary frequency, fatigue, nausea, and weight loss.

### Comparisons of frequency of clinical features in women with early-stage ovarian cancer versus the control group

When women with early-stage ovarian cancer were compared with the control group, 13 features were significantly more common in early-stage cancer, 12 of which had ORs >2.0 (Figure 2). In comparison with the analysis of all cancers, nausea and weight loss were no longer significantly more common whereas urinary incontinence was significantly more common.

## Discussion

### Summary

The frequency of relevant clinical features identified from the EHRs of women before an ovarian cancer diagnosis was much greater when LLM extraction was used than when relying solely on clinical codes. Additionally, associations between most clinical features and ovarian cancer were stronger when LLM-extracted data were used in combination with coded data. For some non-specific clinical features, including change in bowel habit, loss of appetite, weight loss, relying on ICD-coded data alone eliminated associations with cancer that were identified when LLM-extracted data were also used. Given these findings, it is possible that clinical tools that incorporate LLM approaches could help identify patients at elevated risk of an undiagnosed ovarian cancer, including those with lower-risk symptoms and those with early-stage disease, which could support timely investigation and diagnosis.

### Strengths and limitations

A key strength of this study is the use of LLM NLP methods, which enabled improved identification of clinical features. Unlike traditional supervised NLP approaches, which typically require large, task-specific annotated datasets to learn symptom representations, terminology, and negation patterns, LLM-based methods can generalise across heterogeneous clinical language with minimal additional training. In this study, the LLM was able to recognise symptom concepts expressed using varied clinical phrasing and abbreviations, including synonymous and context-dependent expressions that were infrequently or not at all represented in the annotated evaluation set. This robustness to linguistic variation is particularly relevant for primary care and ambulatory records, where documentation styles are highly variable and symptom descriptions are often informal or abbreviated. Such LLM-based extraction may offer greater scalability and portability across clinical settings than conventional rule-based or supervised NLP systems. To the authors’ knowledge, this approach has not previously been applied in the identification of ovarian cancer symptoms and signs. A further strength is that the current study included patients attending a range of clinic types. This is important as women with ovarian cancer can present and be diagnosed via different routes, including the emergency setting, but previous studies generally focused on primary care.^11,14^

The authors of the current study acknowledge several limitations. The criteria for selection of case and control participants restricted eligibility to women with an ambulatory care relationship and evidence of initial workup for ovarian cancer within the UWM system. This minimised inclusion of patients who were seen only for gynaecology specialty services and meant this study was more likely to capture prediagnostic consultation data, but it led to the exclusion of a large proportion of women diagnosed within the UWM system. The study included patients attending a range of clinic types. Although this reflects the healthcare system in the US, where patients may present to different specialties, it is less reflective of healthcare systems where GPs play the central gatekeeping role. It was not possible to distinguish borderline tumours from invasive tumours based on the SEER data obtained. It is possible that borderline tumours may differ from invasive tumours in presenting features. Moreover, this study was designed to examine individual symptoms, and future work would be needed to examine patterns in combinations of symptoms. It was only feasible to annotate a subset of notes (100) for this study. For rare symptoms this meant that there were few annotated incidences that limited precision and interpretability of performance metrics for these symptoms.

A limitation of the LLM-based approach relates to prompt design, particularly the use of expanded synonym lists. Error analysis showed that broader synonym expansion sometimes led to overinference, where the model inferred symptoms from loosely related descriptions. Qualitative error analysis of the annotated evaluation subset suggested that synonym-expanded prompting may increase false-positive extractions for some non-specific symptoms. For example, references to ‘poorer appetite’ were occasionally interpreted as nausea, despite nausea not being explicitly mentioned. In addition, grouping clinically related but non-equivalent terms, such as urinary frequency and urinary urgency, sometimes resulted in incorrect symptom assignment. Non-specific and safety-netting terms, for example, ‘If you develop abdominal pain…’ also contributed to errors. Although a small number of discrepancies were attributable to ambiguous human annotations, overgeneralisation from synonym expansion was the primary contributor to these errors. These findings highlight that LLM-based extraction may overgeneralise when symptom timing or clinical relevance is unclear, which is an important consideration for clinical deployment. A further experimental limitation is that the current study did not compare the approach taken with encoder-based supervised NLP models or rule-based approaches, which may potentially be more straightforward to implement in clinical practice.

### Comparison with existing literature

The current findings support previous research indicating cancer clinical features are frequently recorded in the free text of EHRs and not coded. Price *et al* reported that 38% of symptoms of possible pancreatic and bladder cancer were only recorded in the free text of primary care records.^17^ As per Price *et al*, the current study also found that lower risk symptoms are often recorded in free text and not coded, whereas high risk (‘red flag’) features are commonly coded in patients with cancer. A lung cancer case–control study, which used NLP extraction, found that 20 of 22 possible clinical features of lung cancer were more commonly recorded in the free text than coded.^18^ Recent systematic reviews have not included any studies on NLP extraction of prediagnostic symptoms and signs of ovarian cancer from EHRs.^13,19^

In this study, symptoms and signs, for which an association with ovarian cancer has been reported within the literature were included,^14–16,20^ and found significant associations were present for the majority of features (14 out of 17). Among the multiple symptom-based tools that have been developed to guide clinicians when assessing women for possible ovarian cancer, the Goff Symptom Index is one of the most widely studied and one of the few that has been externally validated.^11^ All six of the symptoms included in the Goff index (increased abdominal size/abdominal distension, abdominal pain, pelvic pain, early satiety/feeling full quickly and loss of appetite/difficulty eating) were significantly more common in the case than the control group in the current analysis. Notably, of these , loss of appetite and early satiety were no longer significantly associated when relying only on coded data.

Importantly, the current study found that a range of symptoms and signs are more common in early-stage cancer than in the control group, which is consistent with the few studies that have examined this,^15,20^ and indicates that early-stage symptomatic detection of ovarian cancer is possible, which would provide greater opportunities for treatment with curative intent.

### Implications for research and practice

The current findings confirm the presence of a signature for ovarian cancer based on clinical features recorded within EHRs. They demonstrate that EHRs contain important information within free text that is not captured by EHR studies relying on codes. Many studies examining presenting features of cancer in primary care, which have informed national guidelines including the National Institute for Health and Care Excellence recommendations, have relied solely of coded data. Considering information within free text could provide a better indication of the risk associated with symptoms in future studies. Clinical decision support tools (CDSTs) for ovarian cancer, other cancers, and other diseases have been developed using coded data from EHRs. Some, such as QCancer^®^ in the UK,^21,22^ can provide automated alerts to clinicians when the risk of cancer (based on codes in the EHR) is above a certain threshold and are used in current clinical practice in some countries. However, the current findings indicate that such tools may miss symptom information in free text that could lead to misclassification bias and have an impact on risk prediction. Given greater identification of relevant symptoms information using LLMs in this study, it is possible that incorporation of free-text symptom information into CDSTs might improve accuracy of individual cancer risk assessment, but further studies are needed to assess this.

This study has focused on the presence of symptoms, however, LLMs can also be used to extract more nuanced information on symptoms such as severity and duration that cannot be obtained with clinical codes alone.^23^ It is possible LLMs applied to clinical records could improve understanding of disease risk and that CDSTs that incorporate LLM extraction approaches might provide a more accurate indication of the risk of cancer and other diseases in individuals. Studies using larger EHR datasets are needed to develop LLM-based CDSTs, evaluate their performance and clinical utility against currently available tools, and examine their potential to reduce delays in referral and diagnosis. To achieve this, greater access to robustly anonymised free-text data for research is required in many countries including the UK.

## Data

Data are available from the authors following appropriate ethical approval.
